# Therapeutic Improvement of Scarring: Mechanisms of Scarless and Scar-Forming Healing and Approaches to the Discovery of New Treatments

**DOI:** 10.1155/2010/405262

**Published:** 2010-08-03

**Authors:** Nick L. Occleston, Anthony D. Metcalfe, Adam Boanas, Nicholas J. Burgoyne, Kerry Nield, Sharon O'Kane, Mark W. J. Ferguson

**Affiliations:** Renovo Group plc, Core Technology Facility, 48 Grafton Street, Manchester M13 9XX, UK

## Abstract

Scarring in the skin after trauma, surgery, burn or sports injury is a major medical problem, often resulting in loss of function, restriction of tissue movement and adverse psychological effects. Whilst various studies have utilised a range of model systems that have increased our understanding of the pathways and processes underlying scar formation, they have typically not translated to the development of effective therapeutic approaches for scar management. Existing treatments are unreliable and unpredictable and there are no prescription drugs for the prevention or treatment of dermal scarring. As a consequence, scar improvement still remains an area of clear medical need. Here we describe the basic science of scar-free and scar-forming healing, the utility of pre-clinical model systems, their translation to humans, and our pioneering approach to the discovery and development of therapeutic approaches for the prophylactic improvement of scarring in man

## 1. Introduction

Anything greater than a superficial injury to the skin of children and adults results in scar formation. Scarring is a major cause of physical and psychological morbidity [1–8]. Whilst various studies have utilised a range of model systems that have increased our understanding of the pathways and processes underlying scar formation, they have not been typically translated to the development of effective therapeutic approaches for scar management. This is evidenced by the fact that despite a number of potential treatment regimens, no single therapy is accepted universally as the standard of care [9–11]. As such, scar improvement still remains an area of clear medical need. Herein, we describe the basic science underlying scar-free and scar-forming healing, the utility and translation of preclinical model systems to humans, and our pioneering approach to the discovery and development of therapeutic approaches for the prophylactic improvement of scarring in man.

## 2. Scar-Free and Scar-Forming Healing

**Figure 1:**
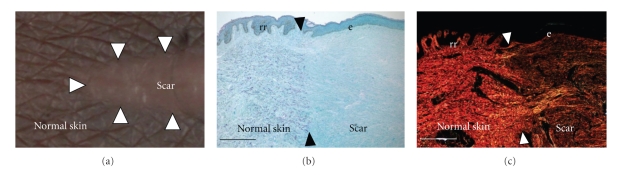
Scarring results from an abnormal deposition and organisation of collagen cutaneous scar in a noncaucasian subject at 12 months following a 1 cm full thickness incision to the inner aspect of the upper arm (a). Histological staining of the excised scar with Van Gieson's stain demonstrating collagen (blue/green) and elastin (purple) staining in the normal skin compared to scar tissue and a normal undulating epidermis with rete ridges in the normal skin compared to a flattened epidermis overlying the scar (b). Picrosirius red staining of the same scar viewed using polarised light (c), illustrating the normal “basket-weave” organisation of collagen in the normal skin resulting in organised light scattering (birefringence) compared to the abnormal organisation of collagen fibres within the scar resulting in a lack of birefringence. Arrowheads indicate the border of normal skin and scar tissue. Scale bars in (b) and (c) are 500 mm. In (b) and (c), rr = rete ridges; e = epithelium.

Scarring and wound healing occur within a spectrum ranging from the ability to completely regenerate tissue in amphibians, through scar-free healing in embryos of different mammalian species, to scar-forming healing in children and adults. From an evolutionary perspective, the scarring response results in rapid replacement of missing tissue and, although suboptimal in terms of appearance and function, results in a reduction in the likelihood of infection and an increased likelihood of organism survival following injury. The ability of organisms to heal wounds without scar formation has nevertheless been demonstrated in the early embryos of a range of mammalian species including mice, rats, rabbits, sheep, pigs, marsupials, and monkeys [12]. Comparison of the architecture of regenerated skin in embryos with that of adults demonstrates that it is the organisation of collagen that is largely responsible for scar formation. Whereas the dermis of embryonic skin is restored to the normal “basket weave” architecture of collagen, in adult scars the collagen is abnormally organised in parallel bundles of fine fibres that are distinct from the normal skin (Figure 1). It is of particular note that there appear to be no major differences between the composition of the dermal tissue of scar-free and scar-forming healing. This indicates that scarring is primarily a failure of the regeneration of the normal skin structure rather than a biochemical problem related to an abnormal composition of the scar tissue [13].

The mechanisms underlying scar-free and scar-forming healing have been studied at the molecular, biochemical, and cellular level. Whilst there are a number of differences that have been identified between healing in the embryo and the adult, many of these are not mechanistically causative. In illustration, the mammalian embryo is surrounded by the sterile aqueous environment of the amniotic fluid, whereas adult wounds are exposed to air and a range of potentially contaminating agents including bacteria and foreign bodies. Originally, it was thought that the sterile aqueous environment provided by the amniotic fluid was important in scar-free healing. However, studies on marsupials such as the opossum, which complete development in their mother's pouch, proved otherwise [14]. In this model, incisional wounds were made in young pouch opossums and at an equivalent embryonic time to mouse embryos in an amniotic environment. Pouch opossums, like embryonic mammals, were found to heal without scarring despite developing outside of a sterile amniotic environment. Following injury to the embryo, the inflammatory response (by virtue of a less than mature immune system) is less marked and differs in terms of the types and number of inflammatory cells that enter the wound [15]. Finally, whilst the profiles and quantities of growth factors and cytokines associated with scar-free healing are often different to those in adult scar-forming healing [16–19], there are only a few of these factors that present themselves as potential therapeutic targets [12, 13, 20]. 

**Figure 2:**
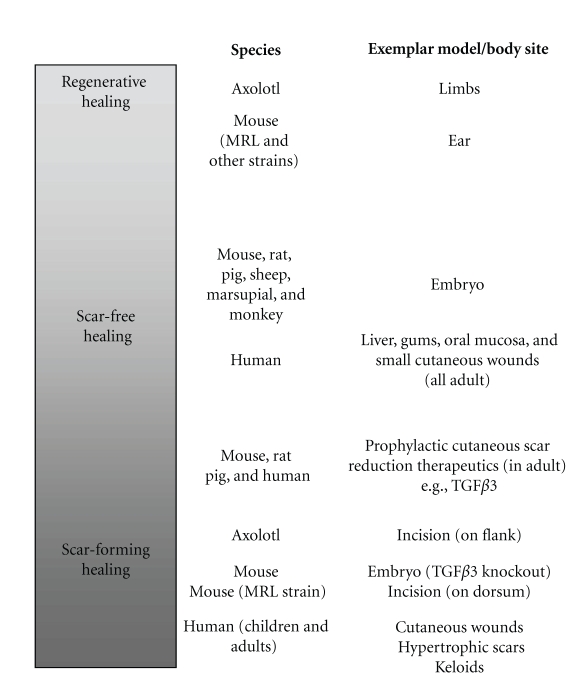
Scar-free to Scar-forming healing in vertebrates represents a continuous spectrum of responses.

Data from our studies and those in the literature demonstrate that the scarring response represents a continuous spectrum of phenotypes in organisms ranging from scar-free through to scar-forming healing (Figure 2). Both scar-free and scar-forming healing can occur in the same animal, for example, an axolotl can regenerate an amputated limb but heals an incisional wound on the flank with a scar; if part of the liver is removed in mammals by hepatectomy, the liver regenerates, whilst stab wounds made to the liver heal with scarring; MRL and other strains of adult mice including athymic nude-*nu* mice regenerate ear wounds, where the absence of T-lymphocytes in wounded ears provides a microenvironment conducive to regeneration of mesenchymal tissues, which is in contrast to wounds made on the dorsum of MRL mice that heal with a scar; penetrating wounds to the cheek of humans heal with scarring of the external cutaneous surface but the oral mucosal surface heals with no discernable scar [13, 21–26]. The likelihood is that tissue repair and regeneration are not that dissimilar and, in fact, share many common mechanisms that differ very subtly. Furthermore, the fact that all mammalian embryos exhibit a regenerative capacity demonstrates that even adult mammals contain the genetic program for regeneration. The above observations are critically important as they demonstrate that organisms retain the ability to heal via a regenerative as well as a scar-forming process, which gives a biological basis for therapeutic modulation of the healing response in adults to reduce scar formation.

## 3. *In Vitro* and *In Vivo* Models to Investigate the Mechanisms of Scarring and Evaluate Potential Treatments

A number of *in vitro* and *in vivo* models have been used to investigate the mechanisms underlying the healing and scarring response. The range of model systems, their uses and limitations are summarised below.

The healing and scarring response consists of a robust series of complex, dynamic and interacting cellular, and molecular processes including haemostasis, the inflammatory response, granulation tissue formation, and remodelling. The functions of the cell types involved in these processes are also regulated by a wide range of extracellular stimuli including growth factors/cytokines as well as interaction with the extracellular matrix [27], which elicit effects by cell surface receptors and a range of intracellular signalling cascades that result in changes in gene and protein expression. Taken together, these events contribute to complex dynamic microenvironments within the injured tissue during healing and scar maturation with which resident and infiltrating cells interact. A number of *in vitro* models have been utilised to investigate the various aspects of the healing and scarring response at the molecular and cellular level and include different cell types (e.g., neutrophils, macrophages, lymphocytes, keratinocytes, melanocytes, fibroblasts, and endothelial cells) and different molecular and cellular processes (e.g., signal transduction, gene expression, proliferation, migration, growth factor production, extracellular matrix production, and remodelling) [28–32]. Whilst *in vitro* models represent applicable systems to study individual components, these systems do not always accurately model the vastly more complex and interactive *in vivo* situation. Typically, we employ *in vitro* systems to further evaluate and refine findings generated from *in vivo* models.

**Figure 3:**
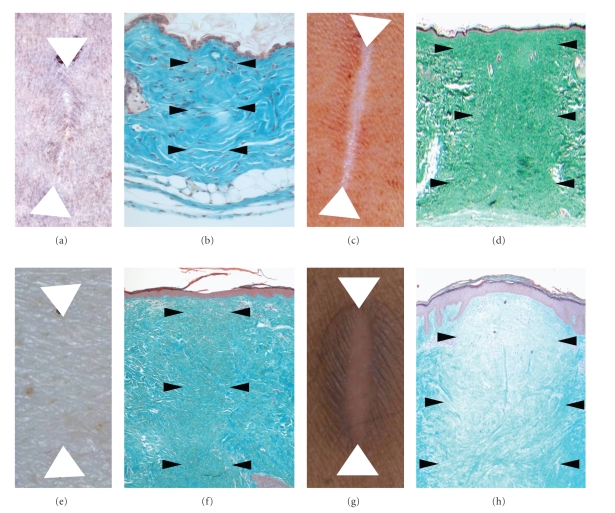
Comparison of Scarring at the Macroscopic and Microscopic Levels between experimental incisional wound models in pre-clinical species and humans. Scarring response in mice 70 days following a 1 cm full thickness incisional wound on the dorsum at the macroscopic ((a) arrowheads indicate ends of original wound) and microscopic ((b) arrowheads indicate scar) levels. Scarring response in rats 84 days following a 1 cm full thickness incisional wound on the dorsum at the macroscopic ((c) arrowheads indicate ends of original wound) and microscopic ((d) arrowheads indicate scar) levels. Scarring response in pigs 168 days following a 1 cm full thickness incisional wound on the dorsum at the macroscopic ((e) arrowheads indicate ends of original wound) and microscopic ((f) arrowheads indicate scar) levels. Scarring response in humans 365 days following a 1 cm full thickness incisional wound on the inner aspect of the upper arm at the macroscopic ((g) arrowheads indicate ends of original wound) and microscopic ((h) arrowheads indicate scar) levels.

**Figure 4:**
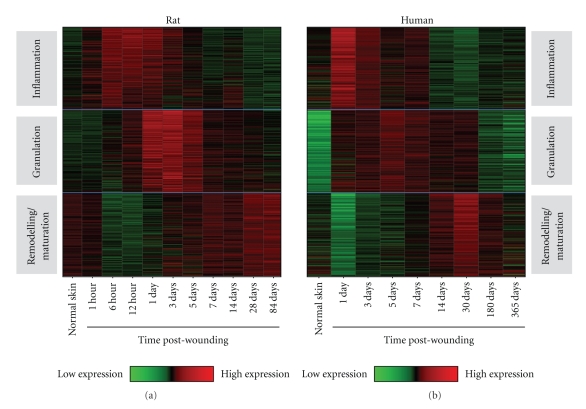
Gene expression in models of incisional wounds and scars in rat and man demonstrate molecular comparability heatmaps of samples of normal skin, wounds, and scars following 1 cm incisional wounds analysed for gene expression using Affymetrix Microarrays comparing the levels and timings of expression of genes involved in the inflammatory, granulation, remodelling, and maturation phases of healing and scarring (examples shown consist of comparison of ~300 genes for each phase).

A number of species including mice, rats, and pigs have been used as potential models of scarring in preclinical studies (Table 1). These studies include the use of excisional and incisional wounds in rodents and pigs, typically with macroscopic and/or microscopic endpoints for scarring, as well as the use of transgenic mice and more recently the Red Duroc pig, which exhibits some of the features of hypertrophic scarring seen in humans [54–56]. Most studies have not systematically compared the molecular, cellular, and tissue responses in these models to those in man. More importantly, most studies have not investigated the translation of therapeutic modulation in preclinical models to that in man. Addressing these issues is key to not only discovering and developing potential therapeutics for humans, but also investigating and understanding their mechanisms of action. We have addressed these issues in a series of extensive longitudinal studies utilising a range of endpoints and technologies both in pre-clinical models and humans. Our studies in mice, rats, and pigs have demonstrated that scars are stable and mature at ≥ 70 days postwounding in mice/rats and ≥6 months in pigs compared to 6 to 12 months in man. Comparison of the macroscopic appearance of these scars and the ability to assess a scar reduction effect within these pre-clinical models demonstrates that next to man, rats scar the worst and represent the most appropriate model (Figure 3). We have also compared the gene expression profiles during the healing and scarring process in these pre-clinical species (up to 30,000 genes per sample per time point; >300 samples; 11 time points) and compared these to profiles in man (Caucasians and Noncaucasians; 30,000 genes per sample per time point; >250 samples; 9 time points). Analyses of the expression of genes involved in all the major phases of healing and scarring have clearly demonstrated molecular comparability, particularly between rat and man, indicating that the major difference between the healing and scarring in these models is time, with humans exhibiting an extended scar maturation phase [50, 51] (Figure 4). In addition, a number of genes/gene pathways have been identified from these studies in rats and man as further potential novel targets for the reduction of scarring in the skin.

## 4. Translation from Pre-Clinical Studies to Clinical Efficacy

As noted above, we have demonstrated that there is significant molecular and cellular comparability between the healing and scarring process in relevant pre-clinical models and in man. Unlike other therapeutic/chronic disease indications, it is important to note that healing and scarring represent an acute biological response that is conserved across species, and the progression of which is somewhat predictable. However, whilst a number of studies have reported therapeutic scar reduction in a variety of pre-clinical models, very few, if any, have demonstrated a translation of these findings to man in suitably designed, controlled, prospective, and randomised clinical trials [9–11]. 

Our approach for the development of therapies has focused on agents for the prophylactic reduction of scarring in man. This involves local administration of these agents to the margins of a wound at the time of surgery that leads to long-term improvements in scarring. The use of prophylactic, regenerative medicines is a novel pharmaceutical approach to scar improvement, and there are a number of challenges associated with this including: designing clinical trials in what is considered a pioneering therapeutic area, developing and validating suitable endpoints for evaluating the effectiveness of a prophylactic drug, where there is no established baseline against which improvements in scarring could be determined (since baseline would otherwise be normal skin before surgery or injury), and patients vary markedly in their propensity for scarring [57, 58]. Our novel approach has been to utilise a within-subject, placebo-controlled, human volunteer model, prior to starting patient studies, not only to establish local drug safety and tolerability but also to investigate a number of other key parameters including: optimal dose(s) and dosing frequency of the drug, evaluation of a variety of relevant endpoints, effects of the drug in subjects with different demographics, for example, sex, race, and age, effects of the drug in different wound types, for example, incisions and excisions. Studies to date have demonstrated that these prospective, double-blind, within-subject designs allow for a relevant and well-controlled approach for determining the proof-of-concept for potential therapies. Since there are no registered pharmaceuticals for the prophylactic reduction of scarring, we have had to pioneer this area in terms of clinical trial design and so have explored a variety of potential surgical models in patient populations to define their appropriateness for demonstration of drug effects. 

We have successfully demonstrated a translation of scar reduction approaches from pre-clinical models to clinical studies, showing clear and robust effects with both ilodecakin (recombinant human interleukin-10, IL-10, Prevascar) in a Phase II clinical trial and with avotermin (recombinant human transforming growth factor beta 3, TGF*β*_3_, Juvista) in extensive Phase II volunteer- and patient-based studies [49, 52, 59]. For example, in three double-blind, placebo-controlled studies, intradermal avotermin (concentrations ranging from 0.25 to 500 ng/100 *μ*L per linear cm wound margin) was administered to both margins of 1 cm, full-thickness skin incisions, before wounding and 24 h later, in healthy men and women [59]. Treatments (avotermin and placebo or standard wound care) were randomly assigned to wound sites by a computer-generated randomisation scheme, and within-participant controls compared avotermin versus placebo or standard wound care alone. Primary endpoints consisted of visual assessment of scar formation at 6 months and 12 months after wounding in two studies, and from week 6 to month 7 after wounding in the third study [59]. All investigators, participants, and scar assessors were blinded to treatment and efficacy analyses. 

In two studies, avotermin 50 ng/100 *μ*L per linear cm significantly improved median score on a 100-mm visual analogue scale (VAS) by 5 mm (range −2 to 14; *P* = .001) at month 6 and 8 mm (−29 to 18; *P* = .0230) at month 12. In the third study, avotermin significantly improved total scar scores at all concentrations versus placebo (mean improvement: from 14.84 mm [95% CI 5.5–24.2] at 5 ng/100 *μ*L per linear cm to 64.25 mm [49.4–79.1] at 500 ng/100 *μ*L per linear cm). Nine [60%] scars treated with avotermin at 50 ng/100 *μ*L per linear cm showed 25% or less abnormal orientation of collagen fibres in the reticular dermis versus five [33%] placebo scars. After only 6 weeks from wounding, avotermin at 500 ng/100 *μ*L per linear cm improved VAS score by 16.12 mm (95% CI 10.61–21.63). 

Similarly, in another Phase II clinical study, intradermal administration of ilodecakin was well tolerated, and at concentrations of 5 ng/100 *μ*L and 25 ng/100 *μ*L per linear cm wound margin, resulted in statistically significant improvements (*P* < .05) in scar appearance with multiple endpoints compared with controls at 12 months postwounding. 

Taken together, the results of these clinical studies have demonstrated that acute, local applications of both avotermin and ilodecakin have the potential to provide an accelerated and permanent improvement in scarring in humans.

## 5. Understanding the Mechanisms of Action of Prophylactic Scar Improvement Therapies

**Figure 5:**
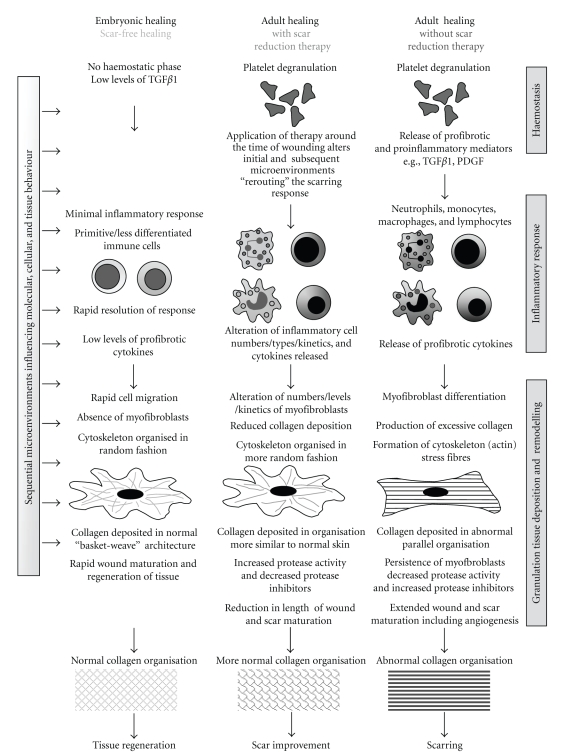
Mechanisms and processes associated with scar-free healing, scar-forming healing and prophylactic scar reduction therapies.

Following cutaneous injury, numerous interacting and dynamic molecular and cellular events are initiated. These include a series of cascades involved in amplification, induction, repression, feed-forward, and feed-back processes that result in a series of sequential and temporal microenvironments within the wound, with which resident cells and those infiltrating the wound interact (Figure 5). The molecular and cellular behaviour of the wound is dependent on the composition of the tissue microenvironment at any one time. Therefore, any alteration of the molecular or functional behaviour of cells (e.g., by appropriate therapeutic modulation) results in changes to subsequent wound microenvironments and ultimately affects the tissue response. In wound healing and scarring, like embryonic development, the system contains a number of pathways exhibiting multiple redundancy which gives robustness to the system. If minor pathways are therapeutically modulated, whilst subsequent microenvironments may be rerouted, they nevertheless result in a scarring phenotype. However, modulation of a major pathway that alters multiple microenvironments synergistically, results in significant alterations and a major “rerouting” of the healing response, leading to the propagation and amplification of a phenotype of improved scar appearance (Figure 5). From our studies in a range of pre-clinical species, we have identified a number of key pathways that are central to generating a scarring response. Our use of human volunteers, in an experimental medicine context, has also rapidly allowed us to confirm which of these identified pathways are relevant in man and hence identify and progress new therapeutics into the clinical arena.

## 6. Summary

The reduction of scarring represents a clear medical need. Currently, there are no registered pharmaceuticals for the prophylactic improvement of scarring, and no single therapy is accepted universally as the standard of care. The spectrum of healing following wounding ranges from the ability to completely regenerate tissue through to the formation of hypertrophic and keloid scars. Importantly, a number of studies have demonstrated that all mammalian organisms retain the ability to heal via both regenerative and scar-forming processes. This is the key in terms of being able to therapeutically modulate the healing response in adults and reduce the severity of subsequent scarring. 

Our approach to the development of therapies has focused on agents for the prophylactic reduction of scarring. This has been significantly aided by our extensive studies, comparing and understanding the molecular processes and scarring phenotypes in pre-clinical models, as well as our pioneering use of human volunteers both in longitudinal scarring studies and in an experimental medicine context which has rapidly allowed us to confirm which of the identified pathways are relevant in man. The translation of findings in the rat pre-clinical model to man has been shown in suitably designed and controlled prospective and randomised clinical trials. 

In terms of the mechanisms of action, the prophylactic administration of scar improvement therapeutics results in significant alterations and a major “rerouting” of the healing response, resulting in the propagation and amplification of a phenotype of improved scar appearance, by virtue of a change in the architecture of the deposited collagen. 

The understanding of the scientific basis of scar-free and scar-forming healing and our pioneering approach to the development of therapies have allowed the identification and progression of new treatments. We have demonstrated that the development of pharmaceuticals for prophylactic scar improvement, that are additive to good surgical technique, is achievable, resulting in new therapies with a sound scientific basis and clear evidence of effectiveness in robust clinical trials.

## Figures and Tables

**Table 1:** *In Vivo* Models Used to Evaluate Scar Improvement Therapies.

Model	Endpoints studied	Utility	Limitations	References
*Transgenic mice * (knockouts and overexpressors; incisions and punch biopsy wounds)	Regeneration, improvements in scarring measured using a range of parameters (gene, protein, histological analysis, tensile strength, macroscopic appearance, etc.). Endpoints typically studied at 14 to 28 days postwounding. Scars stable around day 70.	Initial target identification and validation. Gene modifications may elicit effects on the scarring response, inducing scarless healing or excessive scarring. Incisions most relevant for scarring, punch biopsies relevant for healing endpoints.	Gene deletions/additions can elicit lethal effects and may provide misleading data if compensatory mechanisms due to genetic alteration(s) occur within the animal (also see general comments on mice below).	[22, 23, 33–36]

*Mouse * (incisions and punch biopsy wounds)	Improvements in scarring measured using a range of parameters (gene, protein, histological analysis, tensile strength, macroscopic appearance, etc.). Endpoints typically studied at 14 to 28 days postwounding. Scars stable around day 70 postwounding.	Outbred and inbred strains can be utilised. Gene expression data indicate that molecular processes have a relevant level of comparability to humans. Modulators of the scarring response can be evaluated in the absence of any potentially confounding effects seen in transgenic animals. Incisions most relevant for scarring, punch biopsies relevant for healing endpoints.	Degree of scarring in mice at macroscopic and microscopic levels is significantly less than in humans. Therefore relatively difficult to accurately quantitate improvements with treatments over the normal scarring response. Time points selected for assessment in published studies, for example, 14 to 28 days, are typically during the granulation tissue formation phase prior to formation of a stable scar. As such, these studies do not represent a suitable time point for evaluating the true scar reduction effects of therapies.	[29, 37, 38]

*Rat * (incisions and punch biopsy wounds)	Improvements in scarring measured using a range of parameters (gene, protein, histological analysis, wound width, tensile strength, macroscopic appearance, etc.). Scars stable around 80 days post wounding.	Rats demonstrate comparability to scars in humans (volunteers) at the macroscopic, microscopic, and gene expression levels. Relatively easy to differentiate the effects of scar reducing agents.	Many scientific reagents are geared towards the study of mice and humans, and consequently there are some limitations in terms of reagents (e.g., antibodies for immunocytochemistry) to completely compare all mechanisms to those in man. Most studies use unsuitable time points of <70 days, when scars have not matured/stabilised.	[39–41]

*Rabbit * (incisions and punch biopsy wounds)	Improvements in scarring measured using a range of parameters (gene, protein, histological analysis, macroscopic appearance, etc.). Endpoints typically studied at 20 to 40 days postwounding.	Ear wounds are often used as a model for chronic healing and excessive scarring.	Rarely used to assess normal skin wound healing on the back. Although some features of excessive scarring are modelled, the biological relevance of the ear wounds (involving cartilage) to cutaneous wounds in humans is not completely clear.	[42–44]

*Pig * (minipigs, domestic swine and Red Duroc; incisions, excisions, and punch biopsy wounds)	Improvements in scarring measured using a range of parameters (gene, protein, histological analysis, wound width, tensile strength, macroscopic appearance, etc.). Endpoints typically studied up to 6–12 months postwounding when the scars are stable.	Structure of skin is reported to be most similar to humans. Gene expression data indicates that molecular processes have a relevant level of comparability to humans. Accepted species for wound healing studies. Large or multiple wounds possible due to size. Incisions most relevant for scarring, punch biopsies relevant for healing endpoints. Red Duroc pig is reported to model aspects of hypertrophic scarring in humans.	Degree of scarring in pigs at macroscopic and microscopic levels is significantly less than in humans. Lengthy and costly studies due to timing of relevant endpoints. Red Duroc pigs do not accurately model all relevant aspects of human hypertrophic scars and require significantly long studies and therefore have an associated potentially prohibitive cost. No robust evidence of translation of findings in models to effective therapeutics in prospective, double-blind and well-controlled trials in humans.	[9, 45–48]

*Human volunteers * (incisions, excisions and punch biopsy wounds)	Improvements in scarring measured using a range of parameters (gene, protein, histological analysis, wound width, tensile strength, macroscopic appearance, etc.).	Suitable for demonstrating safety and efficacy. Model highly relevant and translates to patient-based studies. Easy to differentiate the effects of scar reducing agents on a range of clinically and scientifically relevant parameters.	Requirement of suitable infrastructure and expertise.	[13, 49–53]
